# Pericardial Effusion With Tamponade in Lung Cancer Patients During Treatment With Nivolumab: A Report of Two Cases

**DOI:** 10.3389/fonc.2019.00004

**Published:** 2019-01-22

**Authors:** Masahiro Yamasaki, Wakako Daido, Naomi Saito, Kunihiko Funaishi, Takenori Okada, Kazuma Kawamoto, Yu Matsumoto, Naoko Matsumoto, Masaya Taniwaki, Nobuyuki Ohashi, Noboru Hattori

**Affiliations:** ^1^Department of Respiratory Disease, Hiroshima Red Cross Hospital & Atomic-bomb Survivors Hospital, Hiroshima, Japan; ^2^Department of Molecular and Internal Medicine, Graduate School of Biomedical and Health Sciences, Hiroshima University, Hiroshima, Japan; ^3^Department of Respiratory Medicine, Mazda Hospital, Hiroshima, Japan; ^4^Department of Cardiology, Hiroshima Red Cross Hospital & Atomic-bomb Survivors Hospital, Hiroshima, Japan; ^5^Ohashi Clinic, Hiroshima, Japan

**Keywords:** pericardial effusion, tamponade, non-small cell lung cancer, nivolumab, pseudo-progression

## Abstract

**Background:** Nivolumab is an immune checkpoint inhibitor (ICI) that has shown efficacy for treating non-small cell lung cancer and has become a standard therapy for previously treated non-small cell lung cancer. Moreover, immune-related adverse events of ICI therapy are well-known. Malignant pericardial effusions occasionally arise in patients with lung cancer. There have been a few reports of pericardial effusion in non-small cell lung cancer after nivolumab administration. However, the cause of this condition is controversial; the possibilities include serositis as an immune-related adverse event or pseudo-progression.

**Case Presentation:** This report presents two cases of pericardial effusion with tamponade in lung cancer during treatment with nivolumab. Both patients experienced temporal increases in pericardial effusions followed by effusion regression. In one case, nivolumab administration was continued after performance of pericardiocentesis, without an increase in pericardial effusion. In the other case, temporal simultaneous increases in both the pericardial effusion and the primary tumor were detected, followed by simultaneous regression in both the effusion and the tumor. These findings support the fact that the pericardial effusions were caused by pseudo-progression.

**Conclusions:** Pericardial effusion with tamponade can occur in lung cancer patients being treated with nivolumab; moreover, some of these effusions might be caused by pseudo-progression. In the case of putative pseudo-progression, continuation of nivolumab administration might be allowable with strict follow up.

## Background

Nivolumab, an anti-programmed death 1 antibody, is an immune checkpoint inhibitor (ICI) that has shown efficacy for treating non-small cell lung cancer (NSCLC) (1) and, therefore, has become a standard therapy for previously treated NSCLC. Several immune-related adverse events (irAEs) have been reported with nivolumab therapy, such as thyroiditis, pneumonitis, hepatitis, and nephritis (1).

Immune checkpoint inhibitor (ICI) therapy is well-known for affecting the phenomenon of pseudo-progression in solid tumors (2). Pseudo-progression is indicated by a temporary tumor size increase after ICI administration followed by tumor regression, and reflects inflammatory cell infiltration or necrosis (2).

Malignant pericardial effusion occasionally arises in patients with malignant tumors, most commonly cancerous lung tumors (3). Moreover, there have been a few previous reports of pericardial effusion in NSCLC following nivolumab administration (4–8), and some of these occurrences were considered an irAE of nivolumab.

Herein, we report two cases of pericardial effusion with tamponade in lung cancer patients during treatment with nivolumab. The pericardial effusions in the two cases were both malignant. The increases in the effusions were temporary and followed by decreases; therefore, these findings suggest pseudo-progression.

## Case Presentation 1

A 65-year-old man with a 68 pack-year smoking history consulted his primary care physician with the chief complaint of a productive cough. Subsequently, a large mass lesion of his right lung was detected on chest X-ray, and he was referred to our hospital. He was further examined through contrast-enhanced computed tomography (CT), which revealed a mass lesion with a 92-mm diameter, extending from the middle lobe of his right lung to the upper mediastinum, lymphadenopathy of the mediastinum and bilateral neck, swelling of bilateral adrenal grands, intraperitoneal dissemination, and slight pericardial effusion. After further examination, he was diagnosed with adenocarcinoma of the lung, cT4N3M1c, stage IVB (8th edition of the TNM classification for lung cancer). Neither epidermal growth factor receptor (EGFR) mutations nor an anaplastic lymphoma kinase (ALK) gene rearrangement were detected. The patient was treated with four cycles of carboplatin and pemetrexed. Nearly all lesions diminished in size; however, intraperitoneal dissemination worsened.

**Figure 1:**
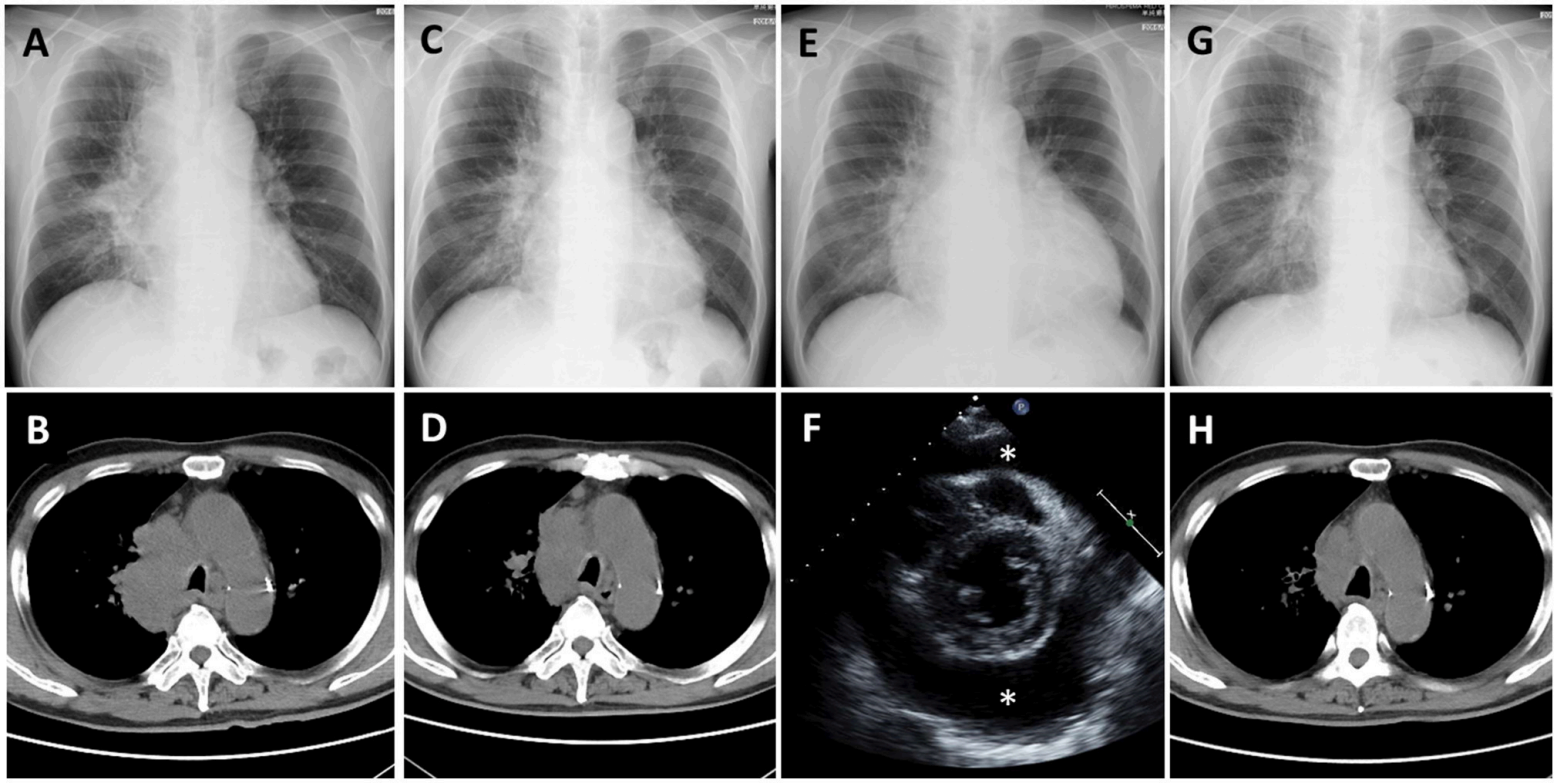
Case 1 **(A,B)** Chest X-ray and computed tomography (CT) before nivolumab administration shows a lung mass from the right mediastinum to the right hilar region. **(C,D)** Chest X-ray and CT after 2 cycles of nivolumab administration shows tumor regression. **(E)** Chest X-ray after 4 cycles of nivolumab administration shows cardiomegaly. **(F)** Echocardiography shows massive pericardial effusion (*: effusion). **(G,H)** Chest X-ray and CT after pericardiocentesis followed by nivolumab administration shows improvement of cardiomegaly and further tumor regression.

Nivolumab therapy was then initiated for the patient (3 mg/kg every 2 weeks) as a second-line therapy. His serum carcinoembryonic antigen (CEA) level before initiation of nivolumab therapy was 143.7 ng/ml; his chest X-ray and CT are presented as Figures 1A,B, respectively. After two cycles of nivolumab administration, the tumor size decreased (Figures 1C,D, respectively). After four cycles of nivolumab administration, he returned to our hospital with the complaint of dyspnea. His blood pressure was 141/85 mmHg, pulse rate was 111/min, and oxygen saturation was 96% on room air. A chest X-ray revealed cardiomegaly, and echocardiography indicated massive pericardial effusion (Figures 1E,F, respectively). He was further diagnosed as having cardiac tamponade. Other irAEs, including myocarditis, were not detected. His serum CEA level was decreased (22.5 ng/ml). He then received pericardiocentesis, and 1,000 ml of bloody effusion was removed. Immediately following this procedure, his condition improved. The pericardial effusion contained 3,025 white blood cells per microliter, and 84% of these cells were lymphocytes. Moreover, cytology revealed adenocarcinoma cells. Despite the fact that nivolumab therapy had not had a positive impact on the pericardial effusion, it had been effective for decreasing the tumor lesions; therefore, the therapy was continued. Corticosteroid treatment was not administered. After five cycles of nivolumab administration following the pericardiocentesis, the pericardial effusion did not recur (Figures 1G,H, respectively); however, intraperitoneal dissemination worsened again, and nivolumab therapy was discontinued. Subsequently, he was treated with several chemotherapies, such as pemetrexed and bevacizumab, gemcitabine, and bevacizumab, as well as *nab*-paclitaxel monotherapy; however, the efficacy of these treatment regimens was limited. Eighteen months after pericardiocentesis, the patient died of lung cancer progression; however, pericardial effusion had not increased.

## Case Presentation 2

A 71-year-old man with a 25 pack-year smoking history visited our hospital with the chief complaints of productive cough and dyspnea. Subsequently, a massive left pleural effusion was detected on chest X-ray. He was then examined through contrast-enhanced CT, which revealed a massive left pleural effusion, a mass lesion with a 36-mm diameter, in the lower lobe of his left lung, and slight pericardial effusion. After further examination, the patient was diagnosed with adenocarcinoma of the lung, cT4N3M1a, stage IVA. Neither EGFR mutations nor an ALK gene rearrangement were detected. He was treated with four cycles of carboplatin and *nab*-paclitaxel, and the treatment was effective for all previously detected lesions; however, multiple brain metastases arose. He then received whole brain irradiation, and these new lesions showed reduction. Subsequently, he was treated with three cycles of pemetrexed as a second-line chemotherapy; however, the primary lesion showed regrowth.

**Figure 2:**
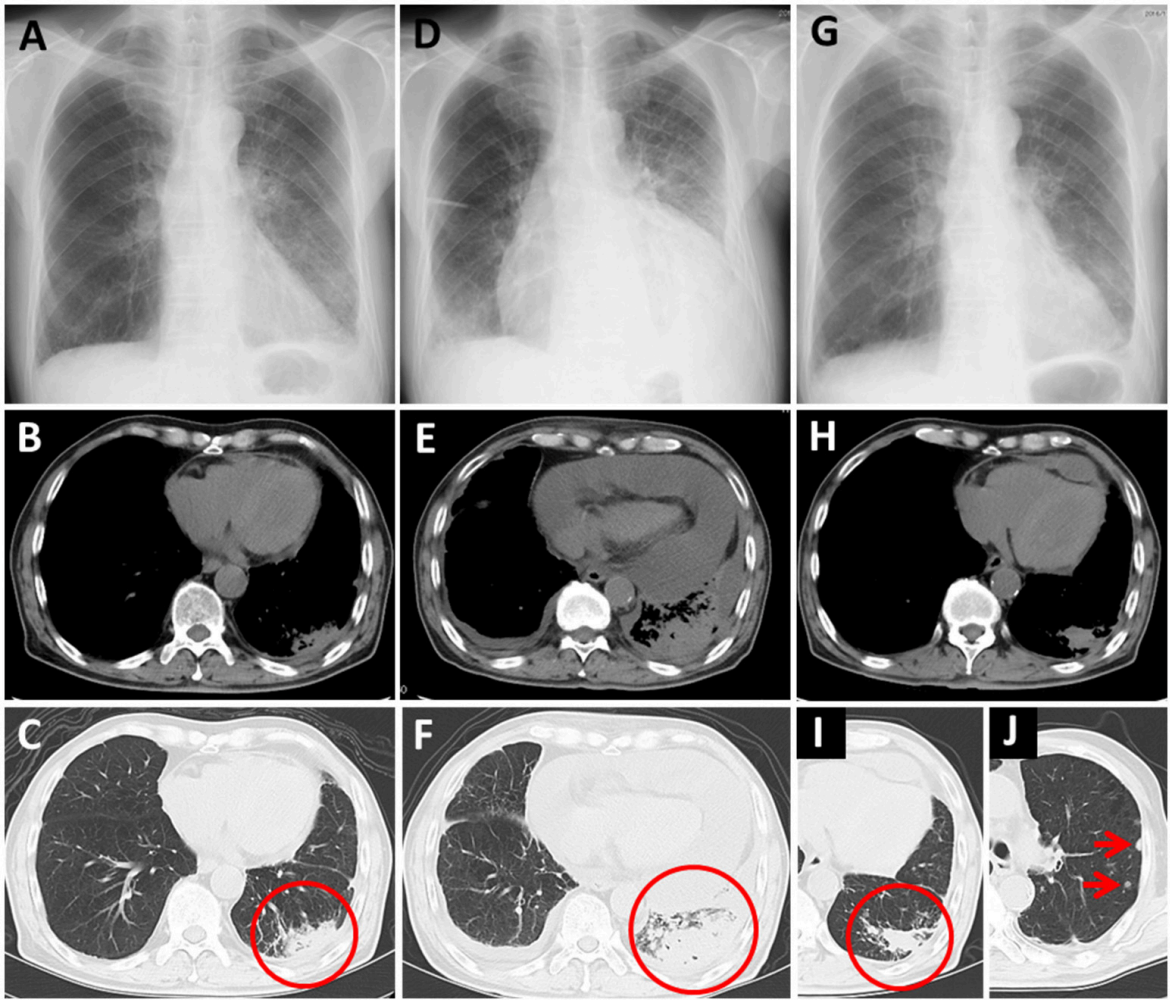
Case 2 **(A–C)** Chest X-ray and computed tomography (CT) before nivolumab administration. Primary lung cancer lesion (circle) and a small amount of pericardial effusion are detected. **(D)** Chest X-ray after nivolumab administration shows cardiomegaly. **(E,F)** Chest CT after nivolumab administration shows massive pericardial effusion and enlargement of the primary lesion (circle). **(G)** Chest X-ray 2 months after the second pericardiocentesis shows no cardiomegaly. **(H–J)** Chest CT 2 months after the second pericardiocentesis shows a decrease in pericardial effusion, reduction of the primary lesion (circle), and a few new intrapulmonary lesions (arrows).

Nivolumab therapy was then initiated for the patient (3 mg/kg every 2 weeks) as a third-line therapy. The chest X-ray and CT before initiation of nivolumab therapy are presented as Figures 2A–C, respectively, (circle: primary lesion), and the serum cytokeratin 19-fragment (CYFRA 21-1) level was 20.7 ng/ml. After two cycles of nivolumab administration, he returned to our hospital with complaints of chest pain and dyspnea. His blood pressure was 95/60 mmHg, pulse rate was 133/min, and oxygen saturation was 89% on 1 L of oxygen delivered by nasal cannula. A chest X-ray revealed cardiomegaly (Figure 2D). Massive pericardial effusion was detected by echocardiography as well as by chest CT (Figure 2E). In addition, the chest CT detected enlargement of the primary lesion (Figure 2F, circle). Other irAEs including myocarditis were not detected. The serum CYFRA 21-1 level was increased (40.7 ng/ml). After he was diagnosed as having cardiac tamponade, he received pericardiocentesis, and 1,400 ml of bloody effusion was removed. The pericardial effusion contained 756 white blood cells per microliter, and 2% of these cells were lymphocytes. Cytology of the effusion detected adenocarcinoma cells. One month after the first pericardiocentesis, pericardial effusion had increased again. Therefore, pericardiocentesis was re-conducted, and 450 ml of pericardial effusion was removed. At this time, the serum CYFRA 21-1 level was decreased (8.8 ng/ml). All anticancer therapy, including nivolumab therapy, was discontinued. Corticosteroid treatment was not administered. Two months after the second pericardiocentesis, a chest X-ray showed no cardiomegaly (Figure 2G), and chest CT showed decreasing pericardial effusion (Figure 2H). Despite the fact that the primary lesion was reduced (Figure 2I, circle), a few new intrapulmonary lesions appeared (Figure 2J, arrows); therefore, the disease was deemed to be progressing. Ten months following the second pericardiocentesis, the patient died of lung cancer progression, without any increase in pericardial effusion.

## Discussion and Conclusions

In this study, we made two important clinical observations. First, we showed that pericardial effusion with tamponade can occur in lung cancer patients during nivolumab treatment, findings similar to those in five previous reports (4–8). In two of these reports, effusions were not malignant and, therefore, were considered irAEs (4, 5), and one of these patients was administered a corticosteroid (5). In another case report, corticosteroid administration was performed without pericardiocentesis or examination of pericardial effusion, and the effusion improved (6). Findings of these reports suggest that the pericardial effusions were irAEs.

Second, some pericardial effusions with tamponade in lung cancer patients during nivolumab treatment might be caused by pseudo-progression. The present two cases showed temporal increasing of pericardial effusions, followed by effusion regression; in addition, the temporal increasing of pericardial effusions were accompanied by tumor marker decreases. In Case 1, nivolumab administration was continued after pericardiocentesis, and pericardial effusion did not increase again. In Case 2, temporal simultaneous increases of the pericardial effusion and the primary tumor were detected, followed by simultaneous regression in both the effusion and the tumor. These findings support the fact that pericardial effusions can be caused by pseudo-progression; however, in Case 2, we could not completely rule out the cause of pericardial effusion as being an irAE, because nivolumab therapy was discontinued after pericardiocentesis. In one previous report, two cases showed increasing malignant pericardial effusions after nivolumab administration, followed by effusion regression; therefore, the pleural effusions were thought to have resulted from pseudo-progression (8).

The cause of pericardial effusion after nivolumab administration is thought to be either nivolumab-induced serositis or pseudo-progression; however, making a distinction between the two causes is difficult. In the present report, Case 1 showed an increased lymphocyte count in the pericardial effusion. One previous report considered a low lymphocyte count in pericardial effusion to indicate pseudo-progression (8), and the present Case 2 also showed a low lymphocyte count; however, the primary indicator of pseudo-progression is infiltration of inflammatory cells, including lymphocytes, into a malignant lesion (2). Therefore, the increase in lymphocytes in one of our patients also suggests that his pericardial effusion originated from pseudo-progression. Comprehensive observation of the courses of pericardial effusion and other lesions is necessary to determine whether or not the cause of pericardial effusion is pseudo-progression. If putative pseudo-progression is determined, as in the present Case 1, continuation of nivolumab administration might be allowable with a strict follow up.

The mechanism behind pseudo-progression's causing pericardial effusion is unclear. An increase in malignant pericardial effusion has been reported to have a close association with vascular endothelial growth factor (VEGF) (9), and VEGF inhibits T-cell development (10). VEGF produced by cancer cells to inhibit T-cell development might cause an increase in malignant pericardial effusion. However, further investigation is needed to determine the mechanism of pericardial effusion following nivolumab administration.

In conclusion, pericardial effusion with tamponade can occur in lung cancer patients during treatment with nivolumab, and some of these effusions might be caused by pseudo-progression. In the case of putative pseudo-progression, continuation of nivolumab administration might be allowable with strict observation and follow up of the patient. We, therefore, suggest that in similar cases, making a determination of pericardial effusion caused by pseudo-progression may improve the outcome of NSCLC patients by allowing continuation of nivolumab therapy.

## Consent for Publication

Informed consent was obtained from the patients for publication of the findings of this case report.

## Author Contributions

MY conceived of the study and drafted the manuscript. WD, NS, KF, and TO collected the data. KK, YM, NM, MT, and NO participated in writing and editing as well as collection of data. NH edited the manuscript. All authors read and approved the final manuscript.

### Conflict of Interest Statement

The authors declare that the research was conducted in the absence of any commercial or financial relationships that could be construed as a potential conflict of interest.

## Abbreviations

ICI: immune checkpoint inhibitor
NSCLC: non-small cell lung cancer
irAE: immune-related adverse event
CT: computed tomography
EGFR: epidermal growth factor receptor
ALK: anaplastic lymphoma kinase
CEA: carcinoembryonic antigen; cytokeratin 19-fragment (CYFRA 21-1)
VEGF: vascular endothelial growth factor.

## References

[1] Kazandjian D Suzman DL Blumenthal G Mushti S He K Libeg M FDA approval summary: nivolumab for the treatment of metastatic non-small cell lung cancer with progression on or after platinum-based chemotherapy. Oncologist (2016) 21:634–42. 10.1634/theoncologist.2015-0507 26984449 PMC4861371

[2] Chiou VL Burotto M. Pseudoprogression and immune-related response in solid tumors. J Clin Oncol. (2015) 33:3541–3. 10.1200/JCO.2015.61.6870 26261262 PMC4622096

[3] He B Yang Z Zhao P Li YJ Wang JG. Cytopathologic analysis of pericardial effusions in 116 cases: implications for poor prognosis in lung cancer patients with positive interpretations. Diagn Cytopathol. (2017) 45:287–93. 10.1002/dc.23671 28139896

[4] Nesfeder J Elsensohn AN Thind M Lennon J Domsky S. Pericardial effusion with tamponade physiology induced by nivolumab. Int J Cardiol. (2016) 222:613–4. 10.1159/000447053 27517649

[5] Kushnir I Wolf I. Nivolumab-induced pericardial tamponade: a case report and discussion. Cardiology (2017) 136:49–51. 10.1016/j.ijcard.2016.08.023 27554835

[6] Shaheen S Mirshahidi H Nagaraj G Hsueh CT. Conservative management of nivolumab-induced pericardial effusion: a case report and review of literature. Exp Hematol Oncol. (2018) 7:11. 10.1186/s40164-018-0104-y 29761026 PMC5941729

[7] Vittorio A Sharma R Siejka D Bhattarai K Hardikar A. Recurrent pericardial effusion while receiving nivolumab for metastatic lung adenocarcinoma: case report and review of the literature. Clin Lung Cancer (2018) 19:e717–20. 10.1016/j.cllc.2018.05.010 29937384

[8] Kolla BC Patel MR. Recurrent pleural effusions and cardiac tamponade as possible manifestations of pseudoprogression associated with nivolumab therapy- a report of two cases. J Immunother Cancer (2016) 4:80. 10.1186/s40425-016-0185-2 27895919 PMC5109681

[9] Karatolios K Pankuweit S Moosdorf RG Maisch B. Vascular endothelial growth factor in malignant and benign pericardial effusion. Clin Cardiol. (2012) 35:377–81. 10.1002/clc.21967 22302718 PMC6652617

[10] Ohm JE Gabrilovich DI Sempowski GD Kisseleva E Parman KS Nadaf S. VEGF inhibits T-cell development and may contribute to tumor-induced immune suppression. Blood (2003) 101:4878–86. 10.1182/blood-2002-07-1956 12586633

